# Monitoring Antiretroviral Therapy in HIV-Infected Children in Resource-Limited Countries: A Tale of Two Epidemics

**DOI:** 10.1155/2011/280901

**Published:** 2010-12-02

**Authors:** Elijah Paintsil

**Affiliations:** Departments of Pediatrics and Pharmacology, Yale School of Medicine, 333 Cedar Street, New Haven, T 06520, USA

## Abstract

Twenty-nine years into the HIV epidemic, several advances have been made; however, there remain several challenges particularly with pediatric HIV in resource-limited countries. The obstacles facing pediatric antiretroviral therapy (ART) delivery in resource-limited countries are multifaceted: lack of health care infrastructure, limited availability of pediatric drug formulations, lack of early HIV diagnostic and monitoring techniques, limited manpower with expertise in pediatric HIV care, limited donor funding, and competing public health priorities with limited health care budget. In this paper, the challenges with various ART monitoring tools in resource-limited countries are discussed. Noninvasive (e.g., patient, clinical events outcome, and adherence) and invasive (e.g., immunologic and virologic) monitoring tools are discussed. Several cheap and technically less complex laboratory tests for monitoring are becoming available. Funding agencies and country programs should invest in validating the use of current technologies to optimize pediatric HIV care in resource-limited countries.

## 1. Introduction

The current state of the HIV epidemic can be likened to the description of the setting of Charles Dickens's novel, “A Tale of Two Cities”—“it was the best of times, it was the worst of times, it was the age of wisdom, it was the age of foolishness, it was the epoch of belief, it was the epoch of incredulity…” Twenty-nine years into the HIV epidemic, several advances have been made; however, there remain several challenges with regard to access and management of antiretroviral therapy (ART), particularly in resource-limited countries. While the birth of an HIV-infected child is rare in resource-rich countries, mother-to-child transmission (MTCT) of HIV continues to fuel the HIV epidemic in resource-limited countries [1]. Two sentinel advances in the pediatric HIV epidemic were (1) an initial 67% reduction in perinatal HIV transmission with the administration of zidovudine (AZT) during pregnancy and peripartum period [2] and (2) a subsequent reduction of perinatal transmission of HIV by 98%-99% in resource-rich countries with the use of highly active antiretroviral therapy (HAART) during pregnancy [3]. Despite these successes, progress has not been uniform worldwide and care for HIV-infected children continues to lag behind. About 2 million of the 2.1 million HIV-infected children live in sub-Saharan Africa, where there is still limited access to antiretroviral drugs even with the unprecedented global effort at scaling up ART [4]. About 1000 children are infected with HIV each day worldwide. At the end of December 2008, only 38% of HIV-infected children less than 15 years of age in resource-limited countries needing ART were on therapy (Table 1) (http://www.who.int/hiv/topics/paediatric/data/en/index.html). The disparity in global coverage of ART,as illustrated in Table 1, underscores the need to scale up pediatric ART delivery. The obstacles facing pediatric ART delivery in resource-limited countries are multifaceted: lack of health care infrastructure, limited availability of pediatric drug formulations, lack of early HIV diagnostic and monitoring techniques, limited manpower with expertise in pediatric HIV care, limited donor funding, and competing public health priorities with limited health care budget [5–7].

The hallmark of HIV infection is progressive CD4+ T cell depletion leading to an increased risk for the development of opportunistic infections, acquired immune deficiency syndrome (AIDS), and death [8–10]. The advent of HAART in 1996 significantly reduced the morbidity and mortality in HIV-infected children in both resource-rich countries [11, 12] and resource-limited countries [13–17]. However, the treatment of HIV infection is a life-long undertaking, and therapeutic benefit can be limited by the evolution of drug-resistant virus and long-term toxicity resulting in treatment failure [18, 19]. There is the need to monitor treatment to early detect and avoid the untoward effects of HAART. In this paper, the successes at monitoring antiretroviral treatment in HIV-infected children in resource-limited countries and the challenges that remain are discussed.

## 2. Monitoring the Response to Antiretroviral Therapy

The goal of HAART is to suppress HIV viral replication and restore immune function. Successful treatment results in virologic suppression, a quantitative increase in the number of CD4+ T cells, and improvement in the clinical well-being of the individual, manifesting as weight gain and resolution or control of opportunistic infections. In resource-limited countries, the World Health Organization (WHO) recommends initiating ART for (i) HIV-infected infants diagnosed in the first year of life, irrespective of CD4 count or WHO clinical stage, (ii) HIV-infected children between 12 and 24 months of age irrespective of CD4+ T cell count or WHO clinical stage, (iii) HIV-infected children between 24 and 59 months of age with CD4+ T cell count of ≤750 cells/mm^3^ or %CD4+ ≤25, whichever is lower, irrespective of WHO clinical stage, (iv) HIV-infected children more than 5 years of age with a CD4+ T cell count of ≤350 cells/mm^3^ (as in adults), irrespective of WHO clinical stage, (v) HIV-infected children with WHO clinical stages 3 and 4, irrespective of CD4 count, and (vi) any child less than 18 months of age who has been given a presumptive clinical diagnosis of HIV infection [20]. Despite the limited armamentarium of first-line antiretroviral drugs in resource-limited countries, national HIV/AIDS programs continue to report good treatment outcomes similar to those in resource-rich countries—with 1 and 2 year survival estimated at 93%–95% and 91%, respectively [14, 15, 21–25]. In resource-rich countries, the standard of care for monitoring treatment in HIV-infected children is the routine laboratory monitoring of CD4+ T cell percentage or count and HIV viral load [26]. In USA, CD4+ T cell percentage or count and HIV viral load are measured at the time of diagnosis of HIV infection and at least every 3-4 months thereafter (http://AIDSinfo.nih.gov/). Due to the lack of accessible and affordable laboratory services, these tests are not routinely available in many resource-limited settings [27]. The WHO highly recommends national programs to develop the laboratory capacity for monitoring ART. However, in the absence of laboratory capacity, clinical parameters are used for monitoring ART. In this paper, the challenges associated with various ART monitoring tools in resource-limited countries are discussed. Noninvasive (e.g., patient, clinical events outcome, and adherence) and invasive (e.g., immunologic and virologic) monitoring tools are discussed.

### 2.1. Patient Monitoring of Antiretroviral Therapy

Direct patient monitoring is the most affordable and cost-effective measure and forms the backbone of clinical care, treatment, and prevention. The WHO has published technical guidelines outlining ART care of infants and children to guide health care delivery programs in resource-limited countries [20]. These guidelines provide a list of essential minimum standards of HIV care and ART monitoring data to be collected at each clinic visit. The collection, collation, and analysis of data on patients over time will be useful for evaluation of both local and national programs. Standardization of the data collection process will also make it easy to identify strengths and weaknesses of programs. An overarching advantage will be the ease of forming ART care coalitions in resource-limited countries to inform best practices parallel to lessons learned from the Pediatric AIDS Clinical Trials Group (PACTG) in resource-rich countries. 

Good record keeping continues to be a perennial problem in most resource-limited countries. Many health care providers are not proficient in basic data management skills, and the few who are proficient in data management are overloaded with work such that data management is not their priority. There is a need for national programs to invest in training data management personnel to assist the providers in collecting, storing, and analyzing data.

#### 2.1.1. Clinical Events Monitoring of Antiretroviral Therapy

Clinical events monitoring provides a noninvasive and low-cost measure for following patients on HAART. Since the ultimate goal of HAART is to restore immune function and halt HIV disease progression, the presence or absence of certain clinical events can be used as surrogates for monitoring the efficacy of HAART. Children starting ART in resource-limited countries are usually severely immunocompromised and very sick [22–25]. The immediate clinical benefit from ART initiation is improvement in the overall well-being and functional status of the patient; subjective and objective measures of these at follow-up visits can be employed to monitor treatment success. Screening questions such as (i) has the patient returned to school or play or is the patient able to engage in activities of daily living without help? (ii) Is the patient ambulating or bedridden? An improvement score could be devised as a quick and subjective assessment of improvement or “return of energy” since starting ART. Using a scale of one to 10, with 10 being “most improvement” and “return of energy” since starting ART, the patient could be asked to score his/her state of health. The answers to these questions will provide a quick measure of the efficacy of ART. However, providers have to be aware of the presenting symptoms of immune reconstitution inflammatory syndrome (IRIS) in their patient cohort as these could confound the functional state assessment [28]. A semiquantitative approach to the clinical assessment that one can explore is to use changes in WHO clinical staging at clinic visits after initiating ART. Studies are needed to determine how long it takes for one to move to the next higher clinical stage after initiating ART and to determine if these changes will accurately predict virologic outcome. 

The majority of children starting ART in resource-limited countries have growth deficiencies. Growth failure in HIV-infected children is a multifaceted problem; it is partly due to the underlying HIV infection itself, with its lack of virologic and immunologic control prior to ART, HIV-associated opportunistic infections, and food deprivation resulting from poverty. Several studies have demonstrated weight gain in children after initiating ART [29, 30]. On average, the weight gain is about 1.8–3.6 kg in the first year of ART. The mean weight-for-age z scores also increase substantially, by about 1 SD from −2 SD or below at baseline [21, 31–34]. In a study of 212 HIV-infected children initiating ART, they continued to catchup in growth and height through the first five years on ART [35]. In these studies, weight gain was significantly associated with virologic control. Where viral load determinations are not feasible, weight gain could be used as part of an algorithm to predict virologic responders.

Clinical algorithms based on signs, symptoms, and simple laboratory assays are being used to identify and treat patients needing ART in resource-limited countries [36, 37]. The Pediatric AIDS Clinical Trials Group (PACTG) 219 Study Team developed and validated a simple Pediatric AIDS Severity Score (PASS) based on baseline weight percentile, WHO clinical stage, symptoms, a general health rating, total lymphocyte count, packed cell volume (hematocrit), and a measure of albumin to guide decisions on initiation of ART in resource-limited countries [38]. The PASS system provided a statistically significant alternative to CD4+ T cell percent and HIV viral load in deciding when to start ART [38]. Such a measure could be adapted in resource-limited countries. Other clinical indices that could provide easy measure of treatment outcome are resolution or decreased frequency or reduction in the severity of HIV-associated illnesses. It has been shown that the incidence of diarrheal diseases, pneumonia, and hospitalization among HIV-infected children decreases substantially on ART [13, 14, 39].

#### 2.1.2. Adherence Monitoring of Antiretroviral Therapy

Adherence to treatment regimens is a prerequisite for the efficacy and durability of any antiretroviral therapy regimens [40, 41]. In resource-limited countries, where second-line regimens are limited, keeping a patient on a first-line regimen as long as possible is an important goal of ART. Patients starting ART should, therefore, be counseled on the need for adherence, and adherence should be monitored at every encounter with the patient. Several studies from resource-limited countries have reported prevalence of adherence among HIV-infected children similar or better than that achieved among those in resource-rich countries [25, 39, 42–45]. There are several measures of adherence, for example, self-report or sophisticated microelectronic monitors that record bottle openings and reconstruct complex pill-taking patterns [46, 47]. Due to resource constraints, self-reporting adherence is used predominantly in resource-limited countries. 

One study compared the relative performance of various low-cost adherence measures: caregiver recall, pill counts at scheduled visits, and unannounced pill counts at home visits. The proportion of patients who achieved perfect adherence (i.e., >95%) was 72%, 89%, and 94% when measured by unannounced pill count, caregiver report, and pill count at scheduled visits, respectively [45]. In a study from South Africa, investigators sought to determine the accuracy of adherence assessments for predicting and detecting virologic failure and to compare the accuracy of adherence-based monitoring with CD4+ T cell monitoring in HIV-infected adults on ART [48]. Pharmacy-based time-to-refill of HAART was used as a measure of adherence [49]. Adherence was calculated as the number of months for which ART claims were submitted to the pharmacy, divided by the number of complete months from ART initiation to the date of study endpoint, and the results multiplied by 100. Adherence values were found to provide statistically significant accuracy for detecting virologic failure at 6 and 12 months compared to CD4+ T cell count changes (AUCs of 0.79 versus 0.68 at 6 months and 0.85 versus 0.75 at 12 months) [48]. This finding implies that in resource-limited countries where CD4+ T cell counts are not readily available, a comprehensive monitoring of pharmacy refill data could be used to identify patients with a high probability of virologic failure. Grossberg et al. determined the validity and utility of pharmacy-based time-to-refill measure of antiretroviral therapy adherence in a cohort of 110 HIV-infected adults [49]. The viral load of study individuals decreased by 0.12 log copies/mL (95% confidence interval [CI] 0.01–0.23 log copies/mL) for each 10% increase in pharmacy-based time-to-refill defined adherence as compared with 0.05 log copies/mL (95% CI: −0.14–0.25 log copies/mL) for the self-reported adherence measure. The key to the validity of using adherence measures as surrogates for monitoring depends on meticulous acquisition, maintenance, collation, and analysis of the data and making pharmacy data available at each patient encounter. Country programs will have to devise a comprehensive and an easy-to-access pharmacy medication refill database. 

Though the importance of adherence is universally recognized, there is no consensus on how to measure adherence. Several programs have adopted adherence measures based on availability of resources. For example, in Malawi, patients are given a 30-day supply of 60 antiretroviral (ARV) pills, and a pill count is carried out at each visit (every 28 days), and patient adherence is said to be >95% if a patient has eight pills or fewer at each visit [41]. Columbia University's MTCT-plus program sites use a 7-day patient recall of number of pills taken, and adherence is categorized according to response as none, very few, about half, most, and all of the pills [20]. The WHO provides a guide for estimating adherence: adherence is said to be “good adherence” (i.e., missing ≤3 doses in a month, ≥95%), “fair adherence” (i.e., missing 4–8 doses in a month, 85%–94%), and “poor adherence” (i.e., missing ≥9 doses in a month, <85%) [20]. It is therefore feasible for all programs to measure adherence as a surrogate of treatment outcome. The irony is that the best measure of adherence is virologic assessment. Therefore, funding agencies and country programs should strive to institute viral load measurement as part of all HIV care programs.

### 2.2. Immunologic Monitoring of Antiretroviral Therapy

CD4+ T cell count remains the single most important parameter in monitoring ART in HIV-infected individuals [26, 50–52]. CD4+ T cell monitoring is more appropriate than virologic monitoring because a decreasing CD4+ T cell count is a better predictor of disease progression [53].  Table 2 illustrates the immunologic and virologic outcomes of antiretroviral therapy in HIV-infected children in resource-limited countries obtained from selected papers published in the last 5 years [22–25, 54–57]. The immunologic outcome of ART in HIV-infected children in resource-limited countries is comparable to that of children in resource-rich countries. However, due to resource constraints and technological challenges, CD4+ T cell count determination is still not available in all HIV care programs in resource-limited countries, and, where available, frequent determinations are not feasible as illustrated by several missing data points in Table 2. In these studies, on average the children doubled their baseline CD4+ T cell count after six months on therapy. The CD4+ T cell count continued to increase at the same rate over the second 6 months and slowed thereafter. The gain in CD4+ T cell count at 18 months on therapy was not very different from that at 12 months. In the Zambian study, where CD4+ T cell values were available for some of the patients at 24 months, there was no significant increase between the 12- and 24- month CD4+ T cell values [24]. Studies of HIV-infected adults have shown that CD4+ T cell recovery reaches a plateau after 4 to 5 years of HAART despite complete viral suppression [58–60]. Could we take advantage of this observation to determine when and how often to monitor CD4+ T cell counts in HIV-infected children on ART in resource-limited countries?

Because of the well-known large natural decline and variation in absolute CD4+ T cell numbers in early childhood [63, 64], the percentage of CD4+ T cell is used for monitoring HIV disease progression in children particularly in those less than 5 years of age [65]. As shown in Table 2, it is interesting to note that the changes in the percentage of CD4+ T cells with treatment are similar to that of absolute CD4+ T cell count. This is consistent with our recent finding that absolute CD4+ T cell count had similar utility as CD4+ T cell percentage in monitoring HIV infection in a pediatric cohort in the US, regardless of age [66]. Others have found that absolute CD4+ T cell counts have less prognostic value in younger children than CD4+ T cell percentage [67]. In resource-limited countries, most low-cost assays available for enumeration of CD4+ T cells mainly provide the absolute counts but not the percentages [27]. There is a need for further studies to examine whether absolute CD4+ T cell counts could be used for children of all ages, especially in situations where available instruments provide only absolute CD4+ T cell counts.

### 2.3. Virologic Monitoring of Antiretroviral Therapy

HIV viral load is a useful tool for initiation and monitoring of ART [51]. It is not a part of the WHO recommendation for routine monitoring of ART in resource-limited countries. HIV-infected children are less likely than infected adults to achieve full viral suppression on ART [68–70]. The cut-off for undetectable viral load differs among studies from resource-limited countries (Table 2); however, the proportion of patients who achieved virologic success is comparable to that in resource-rich countries. Only six of the studies had a viral load for at least two time points. This is most likely due to the cost and the need for sophisticated equipment for determination of viral load. Viral load is a valuable tool for detecting early treatment failure, and it is all the more important in resource-limited countries where second-line regimens are limited. For HIV-infected individuals on ART, it has been found that the CD4+ T cell count and viral load after six months of ART are the strongest predictors of disease progression and death [71, 72]. In resource-limited countries, perhaps viral load determination could also be done less frequently. The critical assay could be the one performed at six months of ART, and the frequency of testing, thereafter, would depend on accessibility, affordability, and the clinical condition of patient.

## 3. Future of Laboratory Monitoring of Antiretroviral Therapy: The Age of Wisdom

Immunologic and virologic measures are the state of the art for monitoring antiretroviral therapy and should be made available in all settings where HIV infection is treated.With the current ARV scale-up campaign, there is an unprecedented call on scientists to develop simpler, more robust, low-maintenance, and cost-efficient laboratory technologies for monitoring antiretroviral therapy in resource-limited countries. Posterity will not forgive modern science if it does not deliver its promise to help mankind in its dire needs [73]. Moreover, the global community sees this as a moral duty, leading to an unusual public-private sector partnership in encouraging and funding the next generation of innovations. 

### 3.1. CD4+ T-Cell Testing

HIV primarily targets CD4+ T cells, and CD4+ T cells play an essential role in HIV pathogenesis [74]. Gut-associated lymphoid tissue (GALT) contains about 60% of the body's total CD4+ T cell pool, and it is an important site of HIV viral replication during acute HIV infection leading to significant CD4+ T cell depletion [75]. Chronic HIV infection affects both quantitative and qualitative function of CD4+ T cells. CD4+ T cell recovery during ART is biphasic: an increase of about 100–200 cells/mm^3^ during the first year of virologic suppression on ART, followed by a gradual increase [76]. Given the central role of CD4+ T cells in HIV pathogenesis, CD4+ T cell determination during the course of HIV disease is one of the most reliable predictors of prognosis [26]. 

Despite the global effort to make CD4+ T cell determination available in all HIV treatment centers, there are still quite a number of centers in resource-limited countries with no access to reliable CD4+ T cell enumeration. The challenge has been to develop a technology easy to operate, able to withstand the tropical environment that could be hostile to equipments, compatible with intermittent electric power delivery, and affordable [77]. Several relatively cheap and technically less complex devices have been developed for CD4+ T cell testing. These include the FACScount system (Becton Dickinson Sciences, California), the Guava Easy CD4 Assay (Guava technologies, Hayward California), the Cyflow (Partec, Germany), and the panleucogating (PGL) CD4 technique [78]. 

Many programs in resource-limited countries are using these affordable technologies; however, not all the devices are designed for pediatric HIV management as they do not measure the percentage of CD4+ T cells. The percentage of CD4+ T cells, rather than the absolute number, has been used as a marker of HIV disease progression in children [65]. This is due to the natural decline and variation in absolute CD4+ T cell numbers in early childhood [63, 64]. There are limited data on the long-term utility of the absolute CD4+ T cell counts to guide treatment changes in pediatric HIV. We recently reported that absolute CD4+ T cell count had similar utility as CD4+ T cell percentage in monitoring HIV infection in a pediatric cohort in USA, regardless of age [66]. There is a need for further research to determine whether absolute CD4+ T cell count can be used in place of CD4+ T cells percentage in managing HIV-infected children of all ages especially in areas where available CD4+ T cell enumeration devices do not measure the CD4+ T cells percentage.

### 3.2. Viral Load Testing

Viral load testing that has been shown to optimize HIV care in resource-rich countries is currently unavailable in most resource-limited countries. HIV RNA assays used in resource-rich countries—Abbott m2000 test, the Roche COBAS Taqman test, bioMérieux NucliSens HIV-1 QT Assay, and Versant HIV-1 RNA 3.0 Assay (bDNA)—are not accessible in resource-limited countries due to costly technical equipment, complex technology, expensive reagents, poor laboratory infrastructure, and prohibitive maintenance cost. Unfortunately, the available commercial assays for viral load determination are very expensive (between $50 and $100 per test) making it unaffordable to many centers in resource-limited countries [79]. CD4+ T cell count may be insensitive for detecting early treatment failure as CD4+ T cells may take several months to drop significantly after virologic failure. Moreover, there are instances of discordant virology and immunologic responses, that is, persistently low or declining CD4+ T cell counts despite complete virologic suppression, or increasing CD4+ T cell count during increasing viremia. Viral load determination is therefore an essential complementary test to CD4+ T cell count [80]. 

There are several cheaper viral load assays being developed and evaluated for use in resource-limited countries. Examples of these assays are the ultrasensitive p24 assay (a signal-amplification boosted ELISA for HIV-1 p24 antigen in plasma after heat-mediated immune complex dissociation), the ExaVir Load reverse transcriptase activity test (an assay that quantify virion-associated reverse transcriptase enzyme), “home-made” real-time PCR HIV-1 RNA assays, the Liat HIV RNA analyzer (http://www.iquum.com/products/analyzer.shtml), and the SAMBA assay (http://www.haem.cam.ac.uk/ddu/). The performances of these assays in comparison to assays used in resource-rich settings have received mixed reviews [81, 82]. Efforts are being made to optimize these assays to improve their sensitivities and specificities. Even when these assays are validated for clinical use, not all HIV clinics will be able to access them locally as resources vary significantly within countries. To make testing available to all clinics, issues surrounding specimen collection, processing, and transport have to be taken into consideration. The use of dried blood spots may be an ideal alternative for specimen collection in rural settings, where there are no laboratories, as it can be transported to testing sites at ambient temperature [81].

### 3.3. What Do We Do in the Interim?

There are preponderance of evidence to suggest that the use of CD4+ T cell enumeration and viral load determination in resource-limited countries is the way to go as the use of clinical algorithms are confounded by the protean of infections and diseases that can masquerade as HIV- or AIDS-associated conditions. The arguments against their routine use are sustained more by consideration of cost, technical expertise, and lack of infrastructure [78]. Country programs should make the effort to adopt available low-cost technologies in clinical care of HIV patients. A question that remains unanswered is: that is it necessary to have frequent determination of CD4+ T cells and viral load as the practice in resource-rich countries is currently? From the data reviewed herein (Table 2), there is little if anything to be gained in measuring CD4+ T cells and viral load more frequently than every 6–12 months. There is a need to come out with appropriate testing intervals which will reduce the overall cost in managing a patient and at the same time sensitive enough to capture patients most likely to fail treatment. Research is needed to investigate and validate monitoring algorithms that utilize all the available tools, that is, more frequent noninvasive measures and invasive measures in a timely and informed fashion.

## 4. Conclusion

Great times and innovative technologies are on the horizon for HIV care, particularly for pediatric HIV care in resource-limited countries. The goal is not to abandon the tried and tested monitoring modalities such as CD4+ T cell count and viral load determinations but to develop technologies that will make these affordable, accessible, and acceptable in resource-limited countries. Funding agencies and country programs should invest in validating the use of current technologies to optimize pediatric HIV care in resource-limited countries.

## Figures and Tables

**Table 1:** Antiretroviral therapy coverage among HIV-infected children less than 15 years of age in resource-limited countries, December 2008.

Geographical region	Number on ART	Number needing ART (range)	Percent of coverage (range)	Percent of total need
Eastern and Southern Africa	195 100	440 000 (340 000–540 000)	44% (36%–57%)	61%
Western and Central Africa	29 800	200 000 (140 000–260 000)	15% (11%–22%)	27%
Latin America	13 700	17 000 (14 000–20 000)	82% (70%–>95%)	2%
The Caribbean	2500	4600 (3400–5800)	55% (43%–72%)	1%
East, South, and South-East Asia	30 000	58 000 (41 000–78 000)	52% (38%–73%)	8%
Europe and Central Asia	4200	4900 (2700–7500)	85% (56%–>95%)	1%
North Africa and the Middle East	400	6700 (3400–11 000)	6% (4%–12%)	1%

Total	275 700	730 000 (580 000–880 000)	38% (31%–47%)	100%

Adapted from (http://www.who.int/hiv/topics/paediatric/data/en/index.html).

**Table 2:** Immunologic and virologic outcomes of antiretroviral therapy in HIV-infected children in resource-limited countries.

Study	1	2	3	4	5	6	7	8	9	10	11	Average of all studies
Number of children	2928	67	151	29	107	212	285	274	67	250	78	—
Country [ref]*	Zambia [24]	India [57]	South Africa [25]	Kenya [54]	Thailand [55]	Cambodia [23]	Haiti [22]	Thailand [56]	Kenya [39]	Uganda [61]	Cote d'Ivoire [62]	—
Median age (years)	6.75	6.28	5.3	8.5	7.7	6	6.3	7	4.4	9.2	6.5	6.7
WHO clinical staging III or IV (%)	72.4	49.3	70.2	62.1	72	64.5	98	65	82	89	na	72.5
Median CD4 count at baseline (cells/mm^3^)	284	225	na	182.3	72	100	608	na	288	272	na	253.9
CD4 gain at 6 months	280	478	na	203	226	na	na	na	210	na	na	279.4
CD4 gain at 12 months	351	516	na	334	332	490	na	na	na	na	na	404.6
CD4 gain at 18^¶^ or 24 months	427^¶^	493^¶^	na	na	532	na	na	na	na	na	na	460^¶^
Median CD4% at baseline	12.9	12	7.4	na	3	6	12	5	5.8	8.6	7.5	8.02
CD4% gain at 6 months	10.8	8	10.2	na	12	na	na	7	9.4	na	4.6	8.86
CD4% gain at 12 months	14.1	11	16.2	na	17	17	10.3	na	na	na	11.1	13.8
CD4% gain at 18^¶^ or 24 months	15.1^¶^	13^¶^	na	na	21	na	na	18	na	na	16	19.5
Median viral load (VL) at baseline (Log)	na	na	na	5.11	5.4	na	5.3	na	6.1	5.3	5.37	5.43
Proportion with undetectable VL at 6 months (%)	na	na	84	~50	53	na	na	na	67	na	52.1	61.2
Proportion with undetectable VL at 12 months (%)	na	na	80.3	na	69	81	56	na	na	74	49.3	68.26
Proportion with undetectable VL at 18 months (%)	na	na	na	na	76	na	na	na	na	na	47.5	61.75
Frequency of CD4 determination (months)	6	3–6	6	3	6	6	6	na	3–6	3–6	6	—

*Reference to papers from which figures were extracted.

^¶^Values available at 18 months.

na: not available.

## References

[1] Paintsil E, Andiman WA (2009). Update on successes and challenges regarding mother-to-child transmission of HIV. *Current Opinion in Pediatrics*.

[2] Connor EM, Sperling RS, Gelber R (1994). Reduction of maternal-infant transmission of human immunodeficiency virus type 1 with zidovudine treatment. *New England Journal of Medicine*.

[3] Yeni PG, Hammer SM, Hirsch MS (2004). Treatment for adult HIV infection: 2004 recommendations of the international AIDS society-USA panel. *Journal of the American Medical Association*.

[4] Report on the global AIDS epidemic; executive summary.

[5] Ginsburg AS, Hoblitzelle CW, Sripipatana TL, Wilfert CM (2007). Provision of care following prevention of mother-to-child HIV transmission services in resource-limited settings. *AIDS*.

[6] Creek T, Ntumy R, Mazhani L (2009). Factors associated with low early uptake of a national program to prevent mother to child transmission of HIV (PMTCT): results of a survey of mothers and providers, Botswana, 2003. *AIDS and Behavior*.

[7] Nkonki LL, Doherty TM, Hill Z, Chopra M, Schaay N, Kendall C (2007). Missed opportunities for participation in prevention of mother to child transmission programmes: simplicity of nevirapine does not necessarily lead to optimal uptake, a qualitative study. *AIDS Research and Therapy*.

[8] Vlahov D, Graham N, Hoover D (1998). Prognostic indicators for AIDS and infectious disease death in HIV-infected injection drug users: plasma viral load and CD4+ cell count. *Journal of the American Medical Association*.

[9] Nishanian P, Taylor JMG, Manna B (1998). Accelerated changes (inflection points) in levels of serum immune activation markers and CD4+ and CD8+ T cells prior to AIDS onset. *Journal of Acquired Immune Deficiency Syndromes and Human Retrovirology*.

[10] Pantaleo G, Graziosi C, Fauci AS (1993). The immunopathogenesis of human immunodeficiency virus infection. *New England Journal of Medicine*.

[11] Gortmaker SL, Hughes M, Cervia J (2001). Effect of combination therapy including protease inhibitors on mortality among children and adolescents infected with HIV-1. *New England Journal of Medicine*.

[12] Gibb DM, Duong T, Tookey PA (2003). Decline in mortality, AIDS, and hospital admissions in perinatally HIV-1 infected children in the United Kingdom and Ireland. *British Medical Journal*.

[13] Fassinou P, Elenga N, Rouet F (2004). Highly active antiretroviral therapies among HIV-1-infected children in Abidjan, Cote d'Ivoire. *AIDS*.

[14] Puthanakit T, Aurpibul L, Oberdorfer P (2007). Hospitalization and mortality among HIV-infected children after receiving highly active antiretroviral therapy. *Clinical Infectious Diseases*.

[15] O’Brien DP, Sauvageot D, Zachariah R, Humblet P (2006). In resource-limited settings good early outcomes can be achieved in children using adult fixed-dose combination antiretroviral therapy. *AIDS*.

[16] O’Brien DP, Sauvageot D, Olson D (2007). Treatment outcomes stratified by baseline immunological status among young children receiving nonnucleoside reverse-transcriptase inhibitor-based antiretroviral therapy in resource-limited settings. *Clinical Infectious Diseases*.

[17] Kline MW, Rugina S, Ilie M (2007). Long-term follow-up of 414 HIV-infected Romanian children and adolescents receiving lopinavir/ritonavir-containing highly active antiretroviral therapy. *Pediatrics*.

[18] Lenert LA, Feddersen M, Sturley A, Lee D (2002). Adverse effects of medications and trade-offs between length of life and quality of life in human immunodeficiency virus infection. *American Journal of Medicine*.

[19] Powderly WG (2002). Long-term exposure to lifelong therapies. *Journal of Acquired Immune Deficiency Syndromes*.

[20] Antiretroviral therapy of HIV infection in infants and children: towards universal access. Recommendations for a public health approach - 2010 revision. http://www.who.int/hiv/pub/guidelines/art/index.html.

[21] Sutcliffe CG, van Dijk JH, Bolton C, Persaud D, Moss WJ (2008). Effectiveness of antiretroviral therapy among HIV-infected children in sub-Saharan Africa. *The Lancet Infectious Diseases*.

[22] George E, Noël F, Bois G (2007). Antiretroviral therapy for HIV-1-infected children in Haiti. *Journal of Infectious Diseases*.

[23] Janssens B, Raleigh B, Soeung S (2007). Effectiveness of highly active antiretroviral therapy in HIV-positive children: evaluation at 12 months in a routine program in Cambodia. *Pediatrics*.

[24] Bolton-Moore C, Mubiana-Mbewe M, Cantrell RA (2007). Clinical outcomes and CD4 cell response in children receiving antiretroviral therapy at primary health care facilities in Zambia. *Journal of the American Medical Association*.

[25] Reddi A, Leeper SC, Grobler AC (2007). Preliminary outcomes of a paediatric highly active antiretroviral therapy cohort from KwaZulu-Natal, South Africa. *BMC Pediatrics*.

[26] Mellors JW, Muñoz A, Giorgi JV (1997). Plasma viral load and CD4+ lymphocytes as prognostic markers of HIV-1 infection. *Annals of Internal Medicine*.

[27] Diagbouga S, Chazallon C, Kazatchkine MD (2003). Successful implementation of a low-cost method for enumerating CD4 + T lymphocytes in resource-limited settings: the ANRS 12-26 study. *AIDS*.

[28] Zampoli M, Kilborn T, Eley B (2007). Tuberculosis during early antiretroviral-induced immune reconstitution in HIV-infected children. *International Journal of Tuberculosis and Lung Disease*.

[29] Nachman SA, Lindsey JC, Moye J (2005). Growth of human immunodeficiency virus-infected children receiving highly active antiretroviral therapy. *Pediatric Infectious Disease Journal*.

[30] Verweel G, van Rossum AM, Hartwig NG, Wolfs TF, Scherpbier HJ, de Groot R (2002). Treatment with highly active antiretroviral therapy in human immunodeficiency virus type 1-infected children is associated with a sustained effect on growth. *Pediatrics*.

[31] Blè C, Floridia M, Muhale C (2007). Efficacy of highly active antiretroviral therapy in HIV-infected, institutionalized orphaned children in Tanzania. *Acta Paediatrica, International Journal of Paediatrics*.

[32] Marazzi MC, Germano P, Liotta G, Buonomo E, Guidotti G, Palombi L (2006). Pediatric highly active antiretroviral therapy in Mozambique: an integrated model of care. *Minerva Pediatrica*.

[33] Walker AS, Mulenga V, Ford D (2007). The impact of daily cotrimoxazole prophylaxis and antiretroviral therapy on mortality and hospital admissions in HIV-infected Zambian children. *Clinical Infectious Diseases*.

[34] Steiner F, Kind C, Aebi C (2001). Growth in human immunodeficiency virus type 1-infected children treated with protease inhibitors. *European Journal of Pediatrics*.

[35] Guillén S, Ramos JT, Resino R, Bellón JM, Muñoz MA (2007). Impact on weight and height with the use of HAART in HIV-infected children. *Pediatric Infectious Disease Journal*.

[36] Farmer P, Léandre F, Mukherjee JS (2001). Community-based approaches to HIV treatment in resource-poor settings. *The Lancet*.

[37] Mekonnen Y, Dukers NHTM, Sanders E (2003). Simple markers for initiating antiretroviral therapy among HIV-infected Ethiopians. *AIDS*.

[38] Patel K, Weinberg GA, Buchacz K, McIntosh K, Dankner WM, Seage GR (2006). Simple pediatric AIDS severity score (PASS): a pediatric severity score for resource-limited settings. *Journal of Acquired Immune Deficiency Syndromes*.

[39] Wamalwa DC, Farquhar C, Obimbo EM (2007). Early response to highly active antiretroviral therapy in HIV-1-infected Kenyan children. *Journal of Acquired Immune Deficiency Syndromes*.

[40] Paterson DL, Swindells S, Mohr J (2000). Adherence to protease inhibitor therapy and outcomes in patients with HIV infection. *Annals of Internal Medicine*.

[41] Harries AD, Gomani P, Teck R (2004). Monitoring the response to antiretroviral therapy in resource-poor settings: the Malawi model. *Transactions of the Royal Society of Tropical Medicine and Hygiene*.

[42] Elise A, France AM, Louise WM (2005). Assessment of adherence to highly active antiretroviral therapy in a cohort of African HIV-infected children in Abidjan, Cote d'Ivoire. *Journal of Acquired Immune Deficiency Syndromes*.

[43] Nyandiko WM, Ayaya S, Nabakwe E (2006). Outcomes of HIV-infected orphaned and non-orphaned children on antiretroviral therapy in Western Kenya. *Journal of Acquired Immune Deficiency Syndromes*.

[44] Bikaako-Kajura W, Luyirika E, Purcell DW (2006). Disclosure of HIV status and adherence to daily drug regimens among HIV-infected children in Uganda. *AIDS and Behavior*.

[45] Nabukeera-Barungi N, Kalyesubula I, Kekitiinwa A, Byakika-Tusiime J, Musoke P (2007). Adherence to antiretroviral therapy in children attending Mulago Hospital, Kampala. *Annals of Tropical Paediatrics*.

[46] Wendel CS, Mohler MJ, Kroesen K, Ampel NM, Gifford AL, Coons SJ (2001). Barriers to use of electronic adherence monitoring in an HIV clinic. *Annals of Pharmacotherapy*.

[47] Miller LG, Hays RD (2000). Measuring adherence to ant i retro viral medications in clinical trials. *HIV Clinical Trials*.

[48] Bisson GP, Gross R, Bellamy S (2008). Pharmacy refill adherence compared with CD4 count changes for monitoring HIV-infected adults on antiretroviral therapy. *PLoS Medicine*.

[49] Grossberg R, Zhang Y, Gross R (2004). A time-to-prescription-refill measure of antiretroviral adherence predicted changes in viral load in HIV. *Journal of Clinical Epidemiology*.

[50] Gulick RM, Mellors JW, Havlir D (1997). Treatment with indinavir, zidovudine, and lamivudine in adults with human immunodeficiency virus infection and prior antiretroviral therapy. *New England Journal of Medicine*.

[51] Hammer SM, Saag MS, Schechter M (2006). Treatment for adult HIV infection: 2006 recommendations of the International AIDS Society-USA panel. *Journal of the American Medical Association*.

[52] O'Brien WA, Hartigan PM, Daar ES, Simberkoff MS, Hamilton JD (1997). Changes in plasma HIV RNA levels and CD4+ lymphocyte counts predict both response to antiretroviral therapy and therapeutic failure. *Annals of Internal Medicine*.

[53] Ghaffari G, Passalacqua DJ, Caicedo JL, Goodenow MM, Sleasman JW (2004). Two-year clinical and immune outcomes in human immunodeficiency virus-infected children who reconstitute CD4 T cells without control of viral replication after combination antiretroviral therapy. *Pediatrics*.

[54] Song R, Jelagat J, Dzombo D (2007). Efficacy of highly active antiretroviral therapy in HIV-1-infected children in Kenya. *Pediatrics*.

[55] Puthanakit T, Oberdorfer A, Akarathum N (2005). Efficacy of highly active antiretroviral therapy in HIV-infected children participating in Thailand’s National Access to Antiretroviral Program. *Clinical Infectious Diseases*.

[56] Puthanakit T, Kerr SJ, Ananworanich J, Bunupuradah T, Boonrak P, Sirisanthana V (2009). Pattern and predictors of immunologic recovery in human immunodeficiency virus-infected children receiving non-nucleoside reverse transcriptase inhibitor-based highly active antiretroviral therapy. *Pediatric Infectious Disease Journal*.

[57] Kumarasamy N, Venkatesh KK, Devaleenol B, Poongulali S, Moothi SN, Solomon S (2009). Safety, tolerability and effectiveness of generic HAART in HIV-infected children in South India. *Journal of Tropical Pediatrics*.

[58] García F, De Lazzari E, Plana M (2004). Long-term CD4+ T-cell response to highly active antiretroviral therapy according to baseline CD4+ T-cell count. *Journal of Acquired Immune Deficiency Syndromes*.

[59] Kaufmann GR, Furrer H, Ledergerber B (2005). Characteristics, determinants, and clinical relevance of CD4 T cell recovery to <500 cells/*μ*L in HIV type 1-infected individuals receiving potent antiretroviral therapy. *Clinical Infectious Diseases*.

[60] Moore RD, Keruly JC (2007). CD4+ cell count 6 years after commencement of highly active antiretroviral therapy in persons with sustained virologic suppression. *Clinical Infectious Diseases*.

[61] Kamya MR, Mayanja-Kizza H, Kambugu A (2007). Predictors of long-term viral failure among ugandan children and adults treated with antiretroviral therapy. *Journal of Acquired Immune Deficiency Syndromes*.

[62] Rouet F, Fassinou P, Inwoley A (2006). Long-term survival and immuno-virological response of African HIV-1-infected children to highly active antiretroviral therapy regimens. *AIDS*.

[63] Wade AM, Ades AE (1998). Incorporating correlations between measurements into the estimation of age-related reference ranges. *Statistics in Medicine*.

[64] Stein DS, Korvick JA, Vermund SH (1992). CD4+ lymphocyte cell enumeration for prediction of clinical course of human immunodeficiency virus disease: a review. *Journal of Infectious Diseases*.

[65] Hughes MD, Stein DS, Gundacker HM, Valentine FT, Phair JP, Volberding PA (1994). Within-subject variation in CD4 lymphocyte count in asymptomatic human immunodeficiency virus infection: implications for patient monitoring. *Journal of Infectious Diseases*.

[66] Paintsil E, Ghebremichael M, Romano S, Andiman WA (2008). Absolute CD4+ T-lymphocyte count as a surrogate marker of pediatric human immunodeficiency virus disease progression. *Pediatric Infectious Disease Journal*.

[67] Dunn D (2005). Use of total lymphocyte count for informing when to start antiretroviral therapy in HIV-infected children: a meta-analysis of longitudinal data. *The Lancet*.

[68] De Rossi A (2004). Virological and immunological response to antiretroviral therapy in HIV-1 infected children: genotypic and phenotypic assays in monitoring virological failure. *New Microbiologica*.

[69] van Rossum AMC, Fraaij PLA, de Groot R (2002). Efficacy of highly active antiretroviral therapy in HIV-1 infected children. *Lancet Infectious Diseases*.

[70] Fraaij PLA, Verweel G, Van Rossum AMC (2005). Sustained viral suppression and immune recovery in HIV type 1-infected children after 4 years of highly active antiretroviral therapy. *Clinical Infectious Diseases*.

[71] Duncombe C, Kerr SJ, Ruxrungtham K (2005). HIV disease progression in a patient cohort treated via a clinical research network in a resource limited setting. *AIDS*.

[72] Chene G, Sterne JA, May M (2003). Prognostic importance of initial response in HIV-1 infected patients starting potent antiretroviral therapy: analysis of prospective studies. *The Lancet*.

[73] Janossy G, Mandy F, O’Gorman MRG (2008). Diagnostics in the shadow of HIV epidemics. *Cytometry B: Clinical Cytometry*.

[74] Schnittman SM, Lane HC, Greenhouse J, Justement JS, Baseler M, Fauci AS (1990). Preferential infection of CD4+ memory T cells by human immunodeficiency virus type 1: evidence for a role in the selective T-cell functional defects observed in infected individuals. *Proceedings of the National Academy of Sciences of the United States of America*.

[75] Veazey RS, DeMaria M, Chalifoux LV (1998). Gastrointestinal tract as a major site of CD4+ T cell depletion and viral replication in SIV infection. *Science*.

[76] Pakker NG, Notermans DW, De Boer RJ (1998). Biphasic kinetics of peripheral blood T cells after triple combination therapy in HIV-1 infection: a composite of redistribution and proliferation. *Nature Medicine*.

[77] Mandy F, Janossy G, Bergeron M, Pilon R, Faucher S (2008). Affordable CD4 T-cell enumeration for resource-limited regions: a status report for 2008. *Cytometry B: Clinical Cytometry*.

[78] Taiwo BO, Murphy RL (2008). Clinical applications and availability of CD4+ T cell count testing in sub-Saharan Africa. *Cytometry B: Clinical Cytometry*.

[79] Katzenstein D, Laga M, Moatti J-P (2003). The evaluation of the HIV/AIDS Drug Access Initiatives in Côte D’ivoire, Senegal and Uganda: how access to antiretroviral treatment can become feasible in Africa. *AIDS*.

[80] Harrigan R (1995). Measuring viral load in the clinical setting. *Journal of Acquired Immune Deficiency Syndromes and Human Retrovirology*.

[81] Fiscus SA, Cheng B, Crowe SM (2006). HIV-1 viral load assays for resource-limited settings. *PLoS Medicine*.

[82] George E, Beauharnais CA, Brignoli E (2007). Potential of a simplified p24 assay for early diagnosis of infant human immunodeficiency virus type 1 infection in Haiti. *Journal of Clinical Microbiology*.

